# Biomechanical comparison between single-row with triple-loaded suture anchor and suture-bridge double-row rotator cuff repair

**DOI:** 10.1186/s12891-020-03654-y

**Published:** 2020-09-24

**Authors:** He-Bei He, Yong Hu, Chuan Li, Cheng-Guo Li, Min-Cong Wang, Hui-Feng Zhu, Zhi-Wen Yan, Cheng-Long Pan, Tao Wang

**Affiliations:** 1 Department of Orthopaedic Surgery, The Fifth Affiliated Hospital of Southern Medical University, Guangzhou City, Guangdong Province China; 2 Department of Orthopaedics, 920 Hospital of Joint Logistics Suppport Force of Chinese People’s Liberation Army, Kunming City, Yunnan Province China; 3 Anatomy Laboratory, Haiyuan College of Kunming Medical University, Kunming City, Yunnan Province China

**Keywords:** Rotator cuff tear, Single-row, Triple-loaded suture anchor, Suture bridge, Biomechanical testing

## Abstract

**Background:**

Numerous biomechanical and clinical studies comparing different techniques for rotator cuff repair have been reported, yet universal consensus regarding the superior technique has not achieved. A medially-based single-row with triple-loaded suture anchor (also referred to as the Southern California Orthopedic Institute [SCOI] row) and a suture-bridge double-row (SB-DR) with Push-Locks have been shown to result in comparable improvement in treating rotator cuff tear, yet the biomechanical difference is unknown. The purpose of the current study was to determine whether a SCOI row repair had comparable initial biomechanical properties to a SB-DR repair.

**Methods:**

Six matched pairs of fresh-frozen cadaveric shoulders with full-thickness supraspinatus tendon tears we created were included. Two different repairs were performed for each pair (SCOI row and SB-DR methods). Specimens were mounted on a material testing machine to undergo cyclic loading, which was cycled from 10 to 100 N at 1 Hz for 500 cycles. Construct gap formation was recorded at an interval of 50 cycles. Samples were then loaded to failure and modes of failure were recorded. Repeated-measures analysis of variance and pair-t test were used for statistical analyses.

**Results:**

The construct gap formation did not differ between SCOI row and SB-DR repairs (*P* = 0.056). The last gap displacement was 1.93 ± 0.37 mm for SCOI row repair, and 1.49 ± 0.55 mm for SB-DR repair. The tensile load for 5 mm of elongation and ultimate failure were higher for SCOI row repair compared to SB-DR repair (*P* = 0.011 and 0.028, respectively). The ultimate failure load was 326.34 ± 11.52 N in the SCOI row group, and 299.82 ± 27.27 N in the SB-DR group. Rotator cuff repair with the SCOI row method failed primarily at the suture- tendon interface, whereas pullout of the lateral row anchors was the primary mechanism of failure for repair with the SB-DR method.

**Conclusion:**

Rotator cuff repair with the SCOI row method has superior biomechanical properties when compared with the SB-DR method. Therefore, SCOI row repair using a medially-based single-row technique with triple-loaded suture anchor is recommended to improve the initial strength in treating full-thickness rotator cuff tears.

## Background

The rotator cuff musculature plays a vital role in maintaining balanced forces that impart mobility and stability of the glenohumeral joint, which leads to pain, restricted motion, and lost productivity when tearing [1]. Tears of the supraspinatus muscle are associated with greater tear displacement, decreased tendon stiffness, and increased regional tendon strains that are caused by affecting the anterior insertion of the rotator cuff cable. Therefore, surgical intervention is always required [2]; however, despite continuous improvement in instrumentation and arthroscopic techniques, the reported risk of rerupture exists and ranged between 20 to 60% [3].

Multiple biomechanical studies have been reported to optimize the healing potential and initial strength of repair in the recent decades [4–12]. Of note, a double-row (DR) repair is considered with increased load-to-failure, improved contact areas and pressures, and decreased gap formation at the healing enthesis compared with single-row (SR) repair [10, 12, 13]. Suture-bridge double-row (SB-DR) with Push-Locks is a construct that simplifies DR fixation by allowing for knotless lateral row fixation using an interference fit of the medial row sutures. SB-DR is considered to be a precursor of “transosseous equivalent” repair, theoretically dominating superior biomechanical properties, greater footprint contact area and pressure [7]. It has been suggested that SB-DR exhibits greater biomechanical characteristics than conventional DR, which in turn is greater than SR [12].

Unlike conventional SR, Dierckman et al. and Dini and Snyder reported that arthroscopic rotator cuff repair using a single-row technique (also referred to as “Southern California Orthopedic Institute [SCOI] row”) consisting of medially-based, triple-loaded anchors augmented with bone marrow vents in the rotator cuff footprint lateral to the repair results in > 90% healing rates and excellent patient reported clinical outcomes [14, 15]. They proposed that a single row with three sutures per anchor yields the strongest possible repair with the least possible tension on the construct; however, verification of such an advantage based on biomechanical studies is limited. It has been suggested that SB-DR and triple-loading SR repairs lead to similar improvement in pain and function with equivalent healing rates [16]. Presumably, the biomechanical properties might also be comparable between SB-DR and triple-loaded SR repairs, but this needs to be verified.

The goal of this cadaver study was to determine the biomechanical properties of full-thickness repair using SCOI row and SB-DR techniques. We hypothesized that the SCOI row repair had comparable initial biomechanical properties to the SB-DR repair.

## Methods

### Specimen preparation

Six matched pairs of fresh-frozen cadaveric shoulders (mean age, 70 ± 3.5 years; range, 67–75 years) from donations to a university anatomy program were included in this study. There was no history of shoulder pathology, injury, or surgery. Donors with underlying musculoskeletal disorders that may affect normal bone or tissue development or function (e.g., muscular dystrophy and multiple sclerosis) were excluded. All specimens were stored at − 20 °C and thawed for 24 h at room temperature before dissection. The tissue was kept hydrated with normal saline during preparation.

All soft tissues were carefully dissected from each specimen, except for the supraspinatus muscle or tendon and the proximal humerus. After dissection, the supraspinatus was sharply detached from the humeral insertion across the entire footprint to simulate a rotator cuff tear. Supraspinatus tendon width and thickness were recorded three times with a digital caliper, averaged, and used to obtain the tendon cross-sectional area (area = width × thickness).

### Repair technique

One supraspinatus tendon from each matched shoulder pair was randomly arranged to be repaired using the SCOI row technique, and the tendon from the contralateral shoulder was repaired using the SB-DR technique.

#### SCOI row repair technique (Fig. 1a)

Two triple-loaded 5.0-mm anchors (Arthrex, Florida, USA) were used for the repair. One anchor was placed 5 mm posterior to the bicipital groove just lateral to the articular margin, and the second anchor was placed 15 mm posterior to the first anchor. Anchors were inserted at 45 degrees. Three simple stiches were placed in a “fan-like” array, spacing the stitches approximately 4–5 mm between passes [17]. Sutures were passed through the tendon using a medium crescent-shaped suture passer. All sutures were tied with locking sliding knots placed over the cuff and followed by three reverse half-hitches on alternating posts (Fig. 2a).

**Fig. 1:**
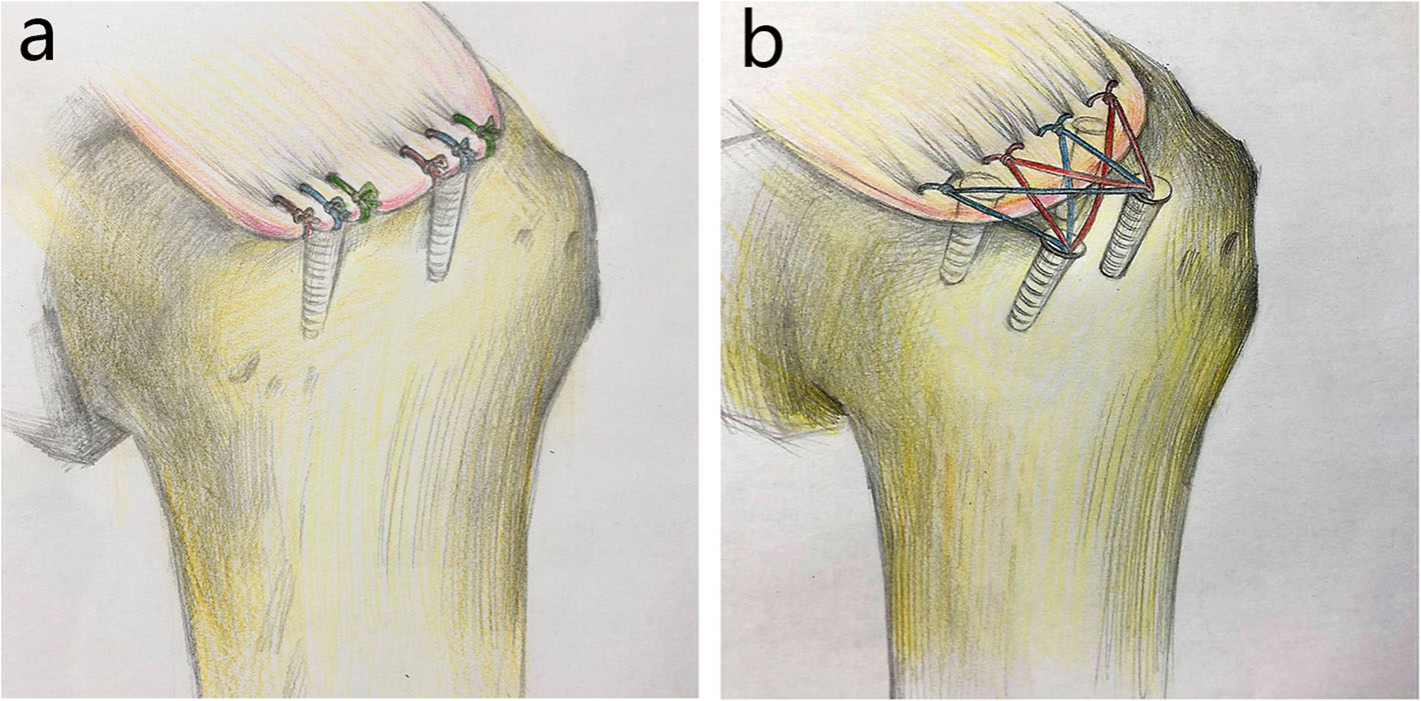
A sketch of two rotator cuff repair techniques: **a.** medially based single-row repair with triple-loaded anchors (SCOI row repair); **b.** Suture-bridge double-row (SB-DR) with PushLocks repair

**Fig. 2:**
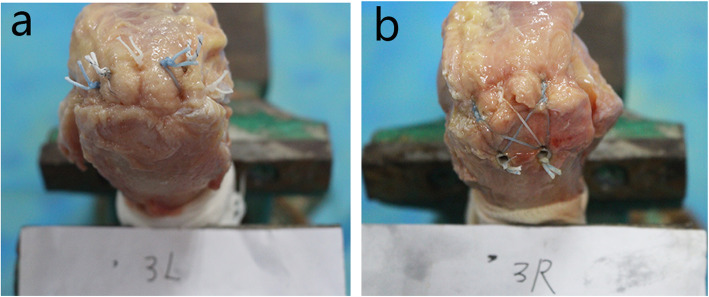
Two rotator cuff repair techniques in cadaver experiments: **a.** medially based single-row repair with triple- loaded anchors (SCOI row repair); **b.** Suture-bridge double-row (SB-DR) with PushLocks repair

#### SB-DR repair technique (Fig. 1b)

Two 5.0-mm medial anchors (Arthrex) with double-loaded sutures were used. The medial row anchors were placed adjacent to the cartilage-footprint junction at 45° to the longitudinal axis of the humerus. The anterior anchor was placed 5 mm posterior to the bicipital groove, while the posterior anchor was placed 12–13 mm posterior to it. Sutures were passed through the tendon using a medium crescent-shaped suture passer, passed for the medial row anchor 5–7 mm apart, and centered about each anchor in the sagittal plane. The sutures were tied using a sliding 3 half-hitch knot. Lateral row fixation was performed with the unilateral 3 sutures end from the medial row anchors with two 4.5 mm Push-Locks instrument (SmithNephew, Hertfordshire, UK). The Push-Lock is a knotless suture fixation device that creates an interference fit of the suture against the bone, which laterally creates a “suture-bridge” stitch (Fig. 2b).

### Biomechanical testing

**Fig. 3:**
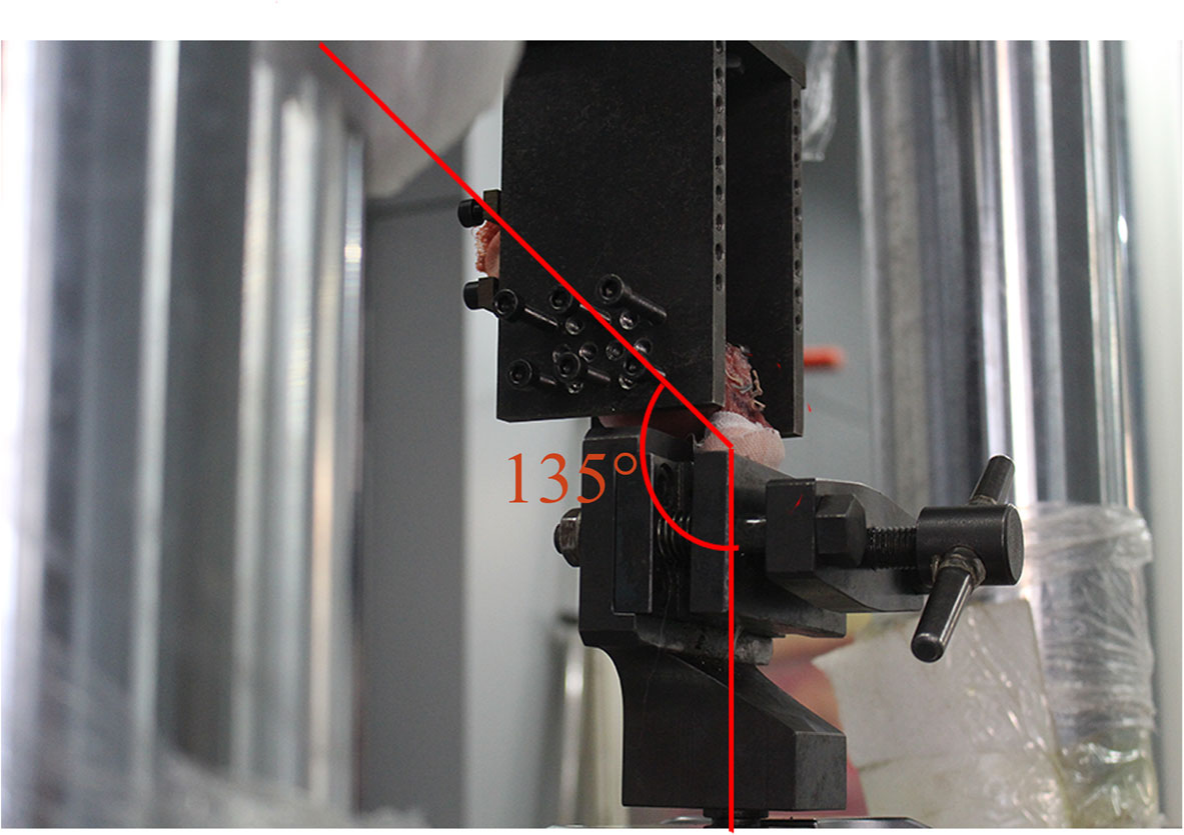
Experimental test setup. Specimens were aligned with an angle of 135 degrees between the muscle line of action and humerus shaft axis

After the repair construct was created, each specimen was mounted into a custom fixture in a material testing machine (Hydraulic Actuator, MTS Systems Corporation, USA). The humeri were transected at the midshaft, then clamped to maintain the humeri at 45 degrees with respect to the test platform (Fig. 3). The humerus was placed at an angle of 135° to the vertical axis, thus allowing tendon testing to approximately re-create the vector of force that would occur after a rotator cuff repair. The tendon was grasped in a specially designed soft-tissue clamp, which had sufficient grip and eliminated tendon slippage.

The biomechanical experimental protocol was adopted from a previous study [6]. A surgical marking pen was used to mark on the tendon for optical tracking of cyclic gap displacement of the repair. Marks were placed on the medial and lateral sides of the repair. During testing, soft tissues were kept hydrated with occasional normal saline spraying. Cyclic loading was performed first. The repaired tendon was preloaded to 10 N at 10 N/s, then held for 5 s. The tendon was cycled from 10 N - 100 N at 1 Hz for 500 cycles [6]. Construct gapping was defined as the displacement between the markers proximal (anatomic medial) to the construct and markers on the bone. Marker displacement was recorded with a digital video system at an interval of 50 cycles. After cyclic loading and a 5-s rest period, constructs were pulled to failure at a rate of 1.5 mm/s. The load was recorded when the construct gap formation reached 5 mm. The ultimate tensile load was defined as the peak force. The failure mechanism for each specimen was also recorded.

### Statistical methods

An a priori power analysis was performed based on the Meisel et al. [11] and Busfield et al. [7] studies, which showed that the differences between the tested rotator cuff repair constructs yielded changes in ultimate failure load of 126 ± 53 N. Using a matched-pairs, two-tailed power analysis (calculated in G*Power 3.192) with the aforementioned differences and standard deviations indicated that 6 matched pairs of speciments can achieve a power of 95%. All statistical analyses were performed using SPSS for Windows (version 23.0). A repeated-measures analysis of variance was performed by comparing the marker displacement during cyclic loading between the two groups. Paired t-tests were used to compare metrics for the matched-pair constructs in loading failure experiment. Differences at a level of *P* < 0.05 were considered statistically significant.

## Results

**Fig. 4:**
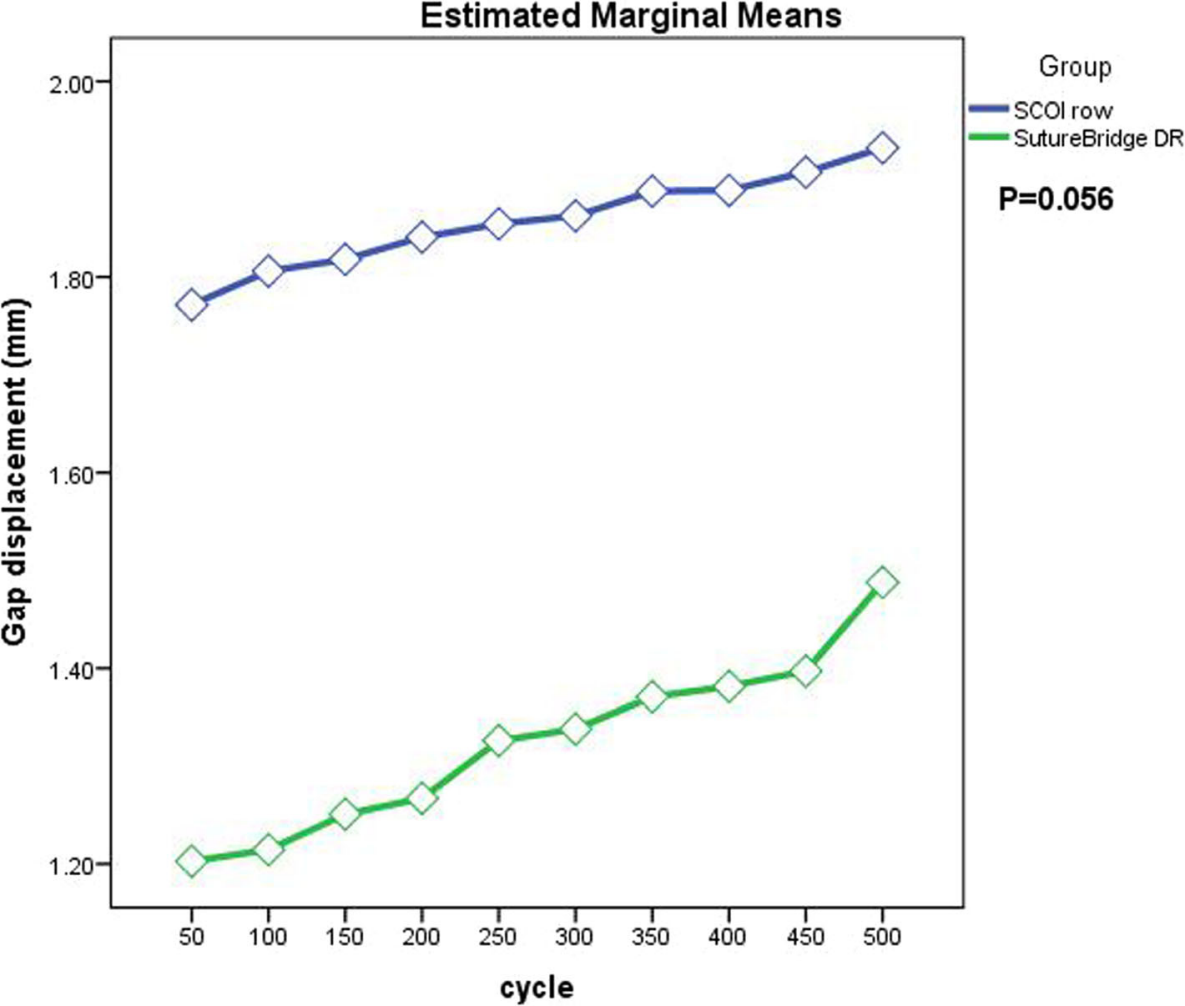
Comparison of gap displacement change between the SCOI row and SB-DR groups. There was no statistical difference between the two groups (*P* = 0.056)

All six pairs of shoulders completed the test. There were no significant differences in the specimen preparation metrics for tendon cross-sectional area between the two groups (*P* = 0.900). Failure was not demonstrated during cyclic loading test in either group. The construct gap displacement was not statistically significant between the SCOI row and SB-DR repairs (*P* = 0.056; Fig. 4). The ultimate gap displacement was 1.93 ± 0.37 mm for the SCOI row group, and 1.49 ± 0.55 mm for the SB-DR group.

The tensile load was 247.16 ± 26.96 N when the construct gap displacement reached 5 mm in the SCOI row group and 196.74 ± 32.28 N in the SB-DR group. The tensile load for 5 mm of elongation was significantly higher in SCOI row repair compared with SB-DR repair (*P* = 0.011, effect size = 1.682, power = 0.9995). The ultimate failure load was also higher in the SCOI row group compared with the SB-DR group and the difference was statistically significant (*p* = 0.028, effect size = 1.118, power = 0.9404). The ultimate failure load was 326.34 ± 11.52 N in the SCOI row group and 299.82 ± 27.27 N in the SB-DR group. The results are shown in Table 1.

**Table 1:** Failure Testing Results, comparing with paired-t test

Cadaver No.	Tendon cross-sectional area (mm^2^)		Load of 5 mm gap formation (N)		Ultimate load (N)	
	SCOI	SB-DR	SCOI	SB-DR	SCOI	SB-DR
1	102.40	98.80	276	230	340	328
2	88.84	93.79	277	170	330	300
3	107.10	99.64	230	170	310	253
4	77.43	88.95	260	244	321	310
5	104.44	96.38	218	187	337	321
6	107.92	115.07	221	178	320	287
Overall mean	98.02	98.77	247.16	196.74	326.34	299.82
Overall SD	12.24	8.87	26.96	32.28	11.52	27.27

**Fig. 5:**
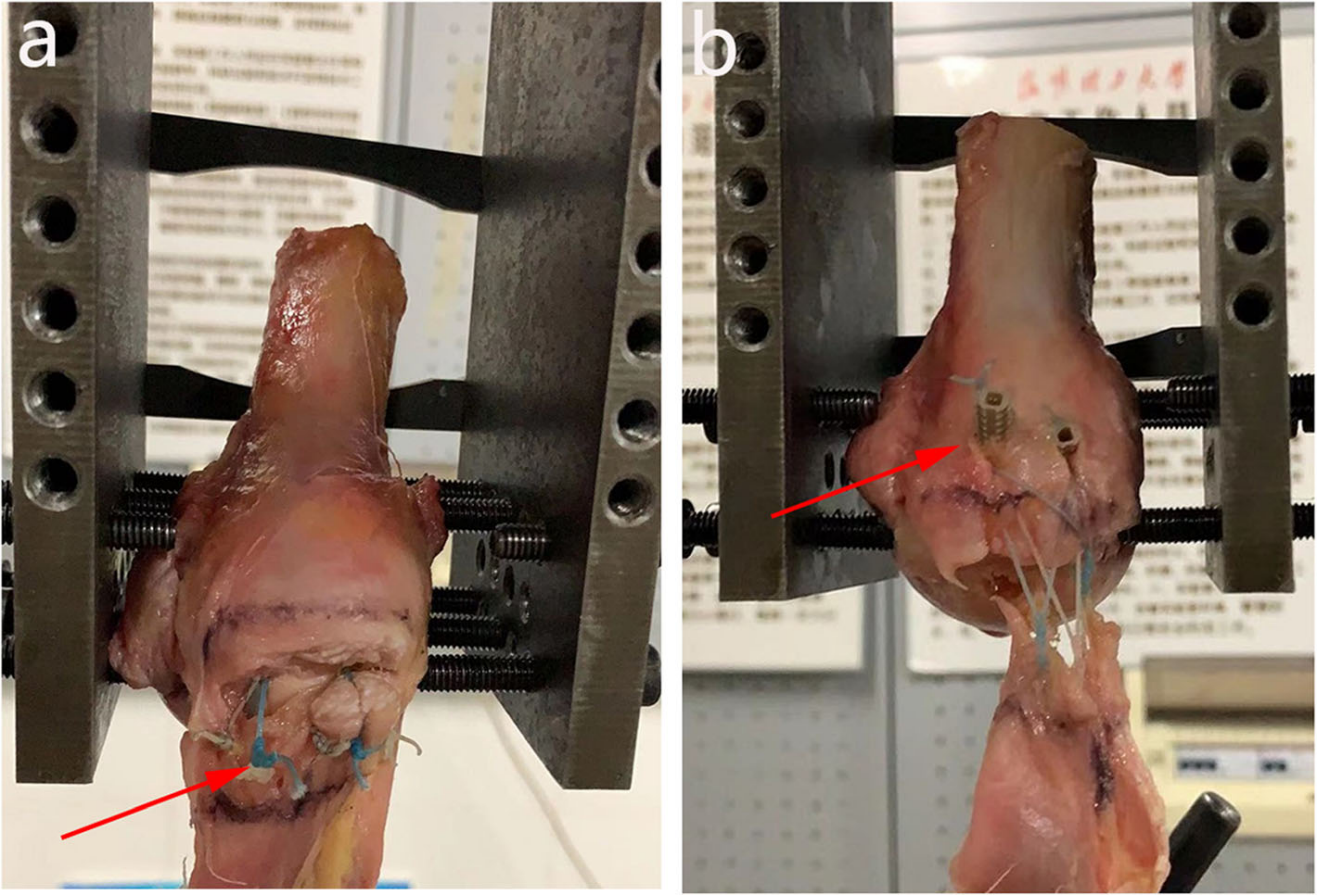
Typical failure mode after rotator cuff repair. **a.** Specimen failure mode for SCOI row repair (suture breakage; red arrow); **b.** Specimen failure mode for SB-DR repair (pullout of lateral row anchor; red arrow)

All 6 six shoulders repaired with the SCOI row construct failed primarily at the suture-tendon interface, with four sutures breaking and 2 sutures cutting through the tendon (Fig. 5a). For shoulders repaired with the SB-DR technique, pullout of the lateral row anchors was the primary mechanism of failure, which occurred in five specimens (Fig. 5b). The remaining specimen failed at the tendon-clamp interface. The conclusions regarding the mode of failure were simple trends because the statistical analysis was not possible to complete given the small numbers in the present study. Thus, the study was not powered to examine modes of failure.

## Discussion

To our knowledge, this is the first study that compared the biomechanical properties of rotator cuff repair using triple-loaded SR (SCOI row) and SB-DR techniques through cyclic loading and failure experiments in cadavers [5, 10, 13, 18, 19]. The immediate repair strength was the major cause of primary repair failure [20]. As a result, superior fixation strength of the repair site was noted using the SCOI row technique, which was inconsistent with our initial hypothesis.

Controversy remains in terms of placing the anchors in a single-row or double-row arrangement [21]. Meier and Meier [22] suggested that DR suture anchor fixation provided significantly stronger initial strength than conventional SR repair, because DR suture anchor fixation consistently reproduces 100% of the original supraspinatus footprint, whereas SR suture anchor fixation reproduces only 46% of the insertion site [23]. Similarly, Kim et al. [13] proposed that DR repair for rotator cuff tear had superior biomechanical properties regarding improved initial strength, stiffness, decreased gap formation and strain over the footprint when compared with a SR repair. The SB-DR technique has been increasingly accepted over the past decade, because the SB-DR technique has several theoretical advantages over the conventional DR repair, such as maximization of the pressurized contact area and decreased tendon strangulation [24, 25]. Quigley et al. [12] reported that the SB-DR repair has better biomechanical characteristics than the conventional DR repair, which in turn is greater than the SR repair.

As summarized by Barber et al. [4], however, the conclusion that SR repairs are more susceptible to gap formation than DR repairs is based on data generated from studies using only one or two sutures in an anchor rather than three. The animal study used by Barber et al. [4] has confirmed that SR with triple-loaded anchors are more resistant to repair failure than conventional and suture-bridge DR repairs. Our human cadaver shoulder study also supported the Barber et al. [4] findings. Similar to Barber et al. [4], a medialized SR repair with triple-loaded suture anchors and tying with simple stitches in a “fan-like” array in each anchor (i.e., the SCOI row method) was performed [14]. As a result, a rotator cuff repair using the SCOI row method was more resistant to tensile load when compared with the SB-DR method.

Our result was inconsistent with the findings of Barber et al. [4] regarding cyclic construct gap displacement. They suggested that the triple-loaded SR constructs were markedly more resistant to gap formation, while we found a comparable result between SCOI row and SB-DR repairs. Lorbach et al. [9] reported result was consistent with our cyclic loading experiment finding. Although the importance of footprint contact area in initial strength of repair and post-operative healing has been increasingly highlighted by advocators of the DR repair [22, 26], Jost et al. [27] showed that it is suture number rather than footprint contact area that determines the strength of a rotator cuff repair. Interestingly, Lorbach et al. [9] further proposed that a SR repair with triple-loaded suture anchors can achieve complete footprint coverage, which was similar to a SB-DR repair. Coons et al. [28] also confirmed that triple-loading sutures in a single anchor are able to increase the tendon repair footprint, and provide superior suture-tendon security subjected to cyclic loading.

Burkhart et al. [29] reported that rotator cuff tears that are repaired with a “tension overload” of a portion of the muscle-tendon units undergo gradual failure with physiologic cyclic loading. Notably, the three sutures are passed as simple stitches in a “fan-like” array in SCOI row repair technique, which evenly distributes the tension and further improve the strength to resist the tensile load. Therefore, the SCOI row method is thought to minimize repair tension and maximize repair strength [14]. Our cyclic loading result provided support for this advantage (Fig. 4). During the cyclic tests, the lower tension enabled the constructs using the SCOI row repair to exhibit a steady and slight increase in gap formation. In contrast, although the overall gap formation was smaller in constructs using the SB-DR repair due to the higher tension, a gross gap displacement change was observed. A rapid increase was initiated at 200 cycle and the last cycle.

The anchors are placed near the articular margin of the greater tuberosity (medial edge of the natural rotator cuff footprint) during the SCOI row method, entering at a 45 degree “tent peg” angle under the subchondral bone a few millimeters lateral to the cartilage [14]. This specific angle was considered to guarantee the strongest fixation of the cuff edge and best resistance to anchor pullout [14]. Our findings led to a consistent conclusion, i.e., pullout of medial anchors was not observed in the failure mode of the SCOI row group. All repairs failed at the suture-tendon interface, with four sutures breaking and two sutures cutting through the tendon. In contrast, pullout of the lateral row anchors was the primary mechanism underlying failure when the rotator cuff was repaired with the SB-DR method. Tingart et al. [30] suggested a relationship between pullout strength of suture anchors and bone mineral density of the tuberosities. Of note, trabecular, cortical, and total bone densities declines as the insertion site moves laterally on the greater tuberosity, thus making the lateral locations weaker anchor insertion sites and more likely to be associated with tendon-bone gap formation [4, 30]. Therefore, the failure location of the lateral row of suture anchors was common in DR constructs [20]. This finding might also partially account for the inferior biomechanical properties of a SB-DR repair compared with SCOI row repair. The current study was not powered to examine modes of failure given the small sample numbers. A well-designed biomechanical and histologic study with sufficient sample size is needed to elucidate the mechanism underlying failure between different rotator cuff repair approaches.

There were several limitations to this study. The fist limitation was the small number of samples (six pairs of fresh-frozen cadavers). Non-pathologic tendons from the cadaver were used in the current study. Pathologic samples with degenerated tendon would be more relevant in a real life situation, because rotator cuff tearing occurs to some extent as a normal degenerative process [31]. Theoretically, the pathologic changes in ruptured tendons may impair the biomechanical properties of the repair site, such as atrophy, fatty infiltration, subtraction of sarcomeres, and profound muscle weakness [32, 33]. Furthermore, the cadaver shoulder is unable to reflect the precise biological performance of an in vivo rotator cuff repair, including tendon quality, blood supply, and process of the healing repair. Modes of failure may be expected to differ based on the variability in tissue quality. Biologic healing and blood supply are also important factors in a rotator cuff repair construct that cannot be addressed in a cadaveric study. In vivo studies are needed to further examine SCOI row repair versus SB-DR repair. The strength of the repair construct was tested at time zero, and the effect of the healing response of a repair over time could not be precisely evaluated in a cadaver model. The current study enrolled Asian cadavers, and the racial difference should not be omitted. A corollary study to determine the difference in terms of contact area, contact pressure, and pressure patterns of the tendon-bone interface between a SCOI row repair and a SB-DR repair is warranted. Finally, only two repair techniques were included in the present study. A study with additional repair techniques is warranted to determine the differences in biomechanical properties.

## Conclusion

In conclusion, rotator cuff repair with the SCOI row method has superior biomechanical properties than the SB-DR technique, as supported by comparable resistance to gap formation during cyclic loading, but higher tensile load for 5 mm of elongation and ultimate load-to-failure. The lower tension of the muscle-tendon units in the footprint might improve the healing rate in a SCOI row repair. Thus, we recommend that the SCOI row method is a reliable option for full-thickness rotator cuff tears.

## Data Availability

The datasets used and/or analyzed during the current study are available from the corresponding author on reasonable request.

## Abbreviations

SCOI: Southern California Orthopedic Institute;
SB-DR: Suture-bridge double-row
DR: Double-row
SR: Single-row
